# Assessment of complementarity of WGCNA and NERI results for identification of modules associated to schizophrenia spectrum disorders

**DOI:** 10.1371/journal.pone.0210431

**Published:** 2019-01-15

**Authors:** Arthur Sant’Anna Feltrin, Ana Carolina Tahira, Sérgio Nery Simões, Helena Brentani, David Corrêa Martins

**Affiliations:** 1 Center for Mathematics, Computation and Cognition, Federal University of ABC (UFABC), Santo André, SP, Brazil; 2 LIM23, Instituto de Psiquiatria, Hospital das Clínicas HCFMUSP, Faculdade de Medicina, Universidade de São Paulo, São Paulo, SP, Brazil; 3 Federal Institute of Education, Science and Technology of Espírito Santo, Serra, ES, Brazil; 4 Instituto de Psiquiatria, Hospital das Clínicas HCFMUSP, Faculdade de Medicina, Universidade de São Paulo, São Paulo, SP, Brazil; 5 National Institute of Developmental Psychiatry for Children and Adolescents (INPD), São Paulo, SP, Brazil; Universidad de Jaen, SPAIN

## Abstract

Psychiatric disorders involve both changes in multiple genes as well different types of variations. As such, gene co-expression networks allowed the comparison of different stages and parts of the brain contributing to an integrated view of genetic variation. Two methods based on co-expression networks presents appealing results: Weighted Gene Correlation Network Analysis (WGCNA) and Network-Medicine Relative Importance (NERI). By selecting two different gene expression databases related to schizophrenia, we evaluated the biological modules selected by both WGCNA and NERI along these databases as well combining both WGCNA and NERI results (WGCNA-NERI). Also we conducted a enrichment analysis for the identification of partial biological function of each result (as well a replication analysis). To appraise the accuracy of whether both algorithms (as well our approach, WGCNA-NERI) were pointing to genes related to schizophrenia and its complex genetic architecture we conducted the MSET analysis, based on a reference gene list of schizophrenia database (SZDB) related to DNA Methylation, Exome, GWAS as well as *copy number variation* mutation studies. The WGCNA results were more associated with inflammatory pathways and immune system response; NERI obtained genes related with cellular regulation, embryological pathways e cellular growth factors. Only NERI were able to provide a statistical meaningful results to the MSET analysis (for Methylation and *de novo* mutations data). However, combining WGCNA and NERI provided a much more larger overlap in these two categories and additionally on Transcriptome database. Our study suggests that using both methods in combination is better for establishing a group of modules and pathways related to a complex disease than using each method individually. NERI is available at: https://bitbucket.org/sergionery/neri.

## Introduction

In linear systems, the performance of the whole is the superposition of the effects of each of its forming parts. In that case, an overall evaluation of the system could be achieved by the separate study of its parts. However, in complex systems, such as gene expression, a global view of the system is different from that obtained by considering only the sum of its parts, or by ignoring the reciprocal influence of its constituent elements. According gene network studies can give us a better view of complex systems [1–4]. As studies in multiples species, and different tissues have shown that co-expressed genes tend to be functionally related [5, 6], co-expression gene network has been widely used [7, 8]. Although there are different problems associated with gene co-expression networks, they represent an excellent tool in the study of complex diseases such as psychiatric diseases [9]. Psychiatric disorders involve changes in multiple genes, and different types of variations as common variation of small effects, very rare or the *de novo* variants intolerant to gene mutation and by uncommon, highly penetrant variants of larger effect as CNVs. All potentially converge in deregulated biological functions. Gene co-expression networks allowed the comparison of different tissues, stages [10] and parts of the brain contributing to an integrated view of genetic variation [11].

Among the diversity of techniques aimed to identify altered network module elements (such as genomic network and biological pathways) that are based on comparison between two groups (i.e. case and control) and complex network analysis, two methods presents appealing results: Weighted Gene Correlation Network Analysis (WGCNA) [12] and Network-Medicine Relative Importance (NERI) [13]. Both methods are based on some Network Medicine hypothesis [14]. WGCNA is a widely used method [12, 15, 16]. Basically it takes as input gene expression data from two groups (for example, control and disease) to build co-expression pairwise correlation matrices (one for each group). This algorithm assumes that the gene co-expression networks present scale-free topology, so the correlation values are powered to increasing natural values (*β*) in order to eliminate edges from the network that do not attend a certain threshold. The final network is the one resulting from the *β* value which maximizes a scale-free independence index (the coefficient of determination of the regression line which best approximates the node degree distribution in log-log scale). Thus, the expression data from case and control groups are clustered using topological overlap measure (TOM) and modules are defined as branches of the resulting clustered tree. Finally, the WGCNA uses the values of TOM from both groups for a module preservation analysis, where it indicates the preservation of the modules across the control and case networks (based on the genes contained on each module). The final result is a list of modules (and its respective genes) ranked by a preservation score. In its turn, NERI [13], integrates gene expression and PPI network data, starting the search for relatively important genes from pre-defined seed nodes (e.g. genes retrieved from association studies (GWAS), using some Network Medicine hypothesis [14] (especially locality, modularity and parsimony) for its network analysis. It also derives two networks (control and disease networks). Each network is a PPI network cutting containing the best shortest paths between each pair of seeds (including the neighbors of the seeds). Each shortest path of a given pair of nodes is evaluated according to the concordance of expression profiles of genes belonging to the considered shortest path. Then, based on each PPI cutting (control and disease), two relative importance scores are obtained for every gene (one for the control cutting and another for the disease cutting). The relative importance score takes into account the number of selected shortest paths between all pair of seeds to which the gene belongs, as well as the respective expression concordance of these paths and the proximity of the considered gene to the seeds. Finally, two ranked lists of genes are outputted: one based on the sum of the relative importance, and another based on the difference between the relative importances.

Thus the WGCNA relying exclusively on the expression data values makes it possible to find all possible genetic relations of the experimented condition whereas NERI is constrained by the PPI scaffold and a set of Seed Genes, what in turn can achieve compelling replication analysis across different datasets. Moreover exclusively co-expression based analysis will better represent genes with a small effect size acting together [17], while NERI permits the usage of *a priori* biological knowledge such as using as seeds rare single nucleotide variations associated to the disorder. Our hypothesis is that both methods are capable of producing compelling results related to different aspects of genetic architecture, and biological processes represented in psychiatric disorders, thus combining both WGCNA and NERI results (WGCNA-NERI), would generate more relevant results than used separately. The main goal of this study is to evaluate the enrichment of biological functions and variations representing the heterogeneous genetic architecture of psychiatric disorders such as schizophrenia by both tools.

By selecting two different gene expression databases related to schizophrenia, we evaluated the biological modules selected by both WGCNA and NERI along these databases; foremost, we conducted a enrichment analysis using DAVID (Database for Annotation, Visualization and Integrated Discover) [18] and KEGG [19], for the identification of partial biological function of each result. To appraise the accuracy of whether both algorithms (as well our approach, WGCNA-NERI) were pointing to genes related to schizophrenia and its complex genetic architecture, we conducted the MSET [20] analysis, based on a reference gene list of the SZDB [21] database, related to CNV (copy number variation), differentially expressed genes (DEG), differentially methylated genes (DMG), exome and GWAS studies.

## Materials and methods

### WGCNA and NERI methods

WGCNA is a widely used method [12, 15, 16]. Basically it takes as input gene expression data from two groups (for example, control and disease) to build co-expression pairwise correlation matrices (one for each group). This algorithm assumes that the gene co-expression networks present scale-free topology, so the correlation values are powered to increasing natural values (*β*) in order to eliminate edges from the network that do not attend a certain threshold. The final network is the one resulting from the *β* value which maximizes a scale-free independence index (the coefficient of determination of the regression line which best approximates the node degree distribution in log-log scale). Thus, the expression data from case and control groups are clustered using topological overlap measure (TOM) and modules are defined as branches of the resulting clustered tree. Until this point, the case and control groups are processed in parallel. Finally, the WGCNA uses the values of TOM from both groups for a module preservation analysis, where it indicates the preservation of the modules across the control and case networks (based on the genes contained on each module). The final result is a list of modules (and its respective genes) ranked by a preservation score.

NERI is a recently developed method which also uses expression data from two groups, but its network construction is based on the integration of PPI network databases, gene expression data and a previously chosen seed genes list (such as based on GWAS studies) [13]. It also derives two networks (control and disease networks). Each network is a PPI network cutting containing the best shortest paths between each pair of seeds (including the neighbors of the seeds). Each shortest path of a given pair of nodes is evaluated according to the concordance of expression profiles of genes belonging to the considered shortest path. Then, based on each PPI cutting (control and disease), two relative importance scores are obtained for every gene—one for the control cutting and another for the disease cutting. The relative importance score takes into account the number of selected shortest paths between all pair of seeds to which the gene belongs, as well as the respective expression concordance of these paths and the proximity of the considered gene to the seeds. Finally, two ranked lists of genes are outputted: one based on the sum of the relative importance, and another based on the difference between the relative importance.

Both methods are based on some Network Medicine hypothesis [2]. WGCNA strongly relies on the assumption that gene co-expression networks tend to follow a scale-free topology, where there are some hubs (highly connected nodes) which are mostly essential to maintain the system functioning (some problem with a hub gene or a hub protein might be lethal). It also assumes the disease module hypothesis (gene products associated to the same disease phenotype tend to participate in a same cluster). NERI, in its turn, assumes the local hypothesis (gene products associated with similar diseases are likely to interact with each other), network parsimony (shortest paths between two known disease genes often coincide with pathways associated to the disease) and also, the disease module hypothesis.

**Fig 1:**
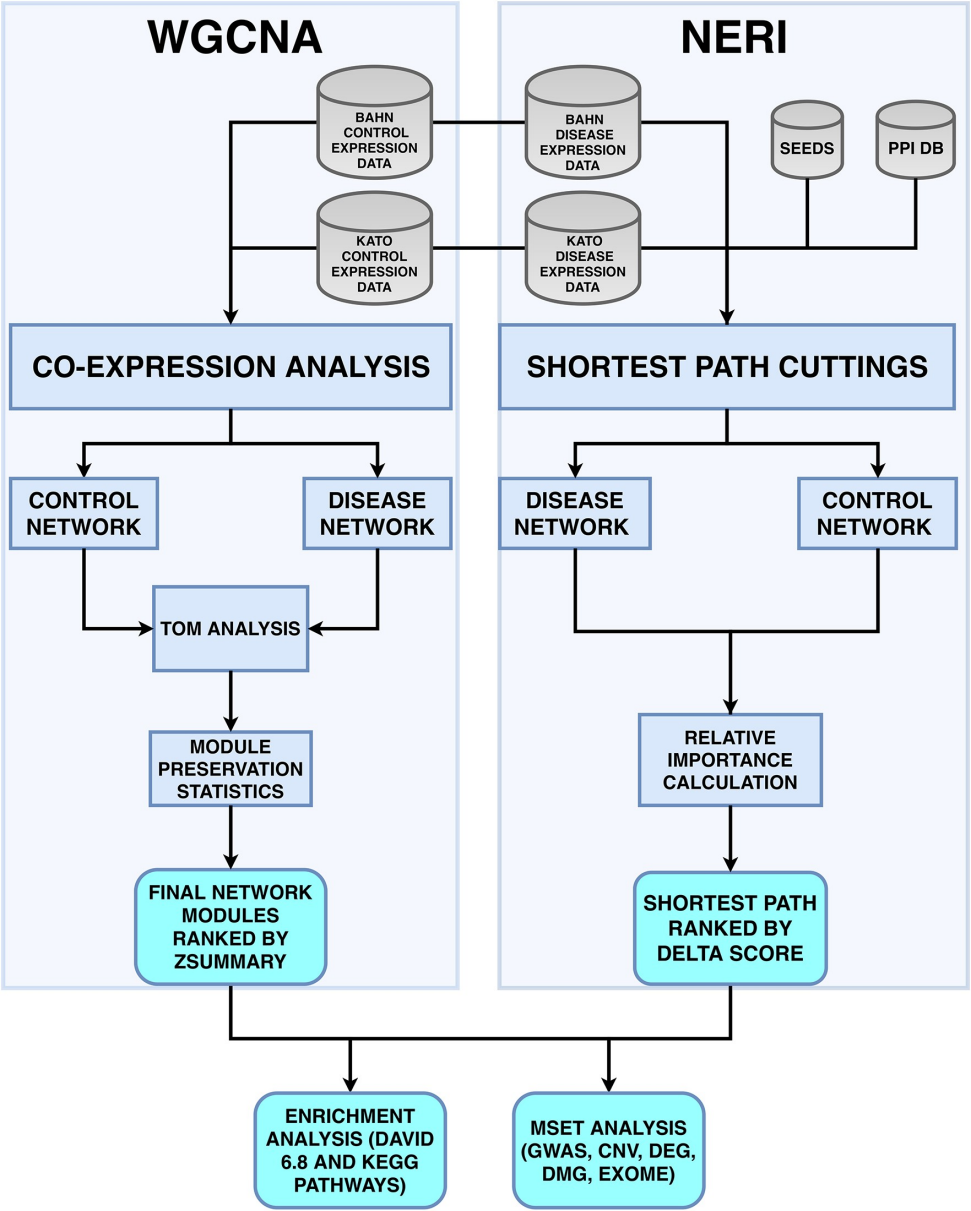
Fluxogram with the pipeline for both WGCNA and NERI. Using the same datasets (BAHN and KATO), both methods utilizes different types of network analysis based on case and control groups, co-expression analysis and Network Medicine’s concepts. WGCNA uses the expression dataset values for the creation of each case and control networks; NERI method integrates different types of data (such as Seeds and PPI databases), which are necessary for create a case and control networks, based on the analysis of all shortest path between all pairs of seed genes in these networks. Our approach, WGCA-NERI consist in the combination of WGCNA’s least preserved module and NERI’s Δ′ score lists. With these results (for both BAHN and KATO dasets), we perfomed an enrichment analysis (using DAVID ver.6.8 and KEGG databases), an MSET analysis, comparing the overlap of these genes with lists of data (CNV, DEG, DMG, exome and GWAS) related o schizophrenia from the SZDB database—and finally, we identified the hub genes from both WGCNA and NERI results and its overlap with the MSET results.

Even if the same gene expression dataset is used, both tools would probably lead to different results, since WGCNA relies only on gene expression data, while NERI uses a combination of PPI networks, gene expression and prior knowledge of disease association (seeds). Considering that NERI is a recently published method; therefore, lacks comparison between this method and other methods—besides standard techniques such as Random Walk with Restart and DADA [13, 22]—and that WGCNA is still widely used, it is important to analyze the similarities and differences of the results of these methods, specially regarding the replication using different gene expression datasets with control and disease groups. We propose an approach to analyze the similarity and differences of both results, and specially if they can be used to complement each other to increase the possibility of gene discovering associated to a complex disease like schizophrenia, consisting in combining the lists obtained in WGCNA’s least preserved module and NERI’s Δ′ score (WGCNA-NERI). Fig 1 shows an overview of the general pipeline used in this study.

### Gene expression datasets

We used two gene expression datasets of *post-mortem* prefrontal cortex samples related to Schizophrenia (available on *Stanley Neuropathology Consortium Integrative Database—SNCID*, available on http://sncid.stanleyresearch.org/Default.asp): BAHN (67 samples) and KATO (69 samples). BAHN and KATO datasets were obtained from the same biological tissues processed on two different experiments. Both datasets are available on https://www.stanleygenomics.org/stanley/standard/studyDetail.jsp?stud_id=3 and https://www.stanleygenomics.org/stanley/standard/studyDetail.jsp?study_id=7, respectively. Demographic summary statistics of each study is shown in details in Table 1.

**Table 1:** Clinical Demographic Statistics from *post-morten* encephalic tissue of prefrontal cortex (BA46A). The chip hgu133a—Affymetrix Human Genome U133 Set—were used in all three databases. All p-values were calculated according to data distribution *X*^2^-test, Student or Mann-Whitney.

Clinical variables	KATO (n = 69)			BAHN (n = 67)		
	CTL (n = 34)	SCZ (n = 35)	P-value	CTL (n = 33)	SCZ (n = 34)	P-value
Age	Mean:44.03 Std:7.68	Mean:42.60 Std:8.38	0.3928	Mean:43.48 Std:7.30	Mean:42.50 Std:8.48	0.6172
Left Brain	Frozen:15 Fixed:19	Frozen:17 Fixed:18	0.9	Frozen:15 Fixed:18	Frozen:16 Fixed:18	1
PMI	Mean:29.62 Std:12.78	Mean:31.40 Std:15.32	0.6481	Mean:29.70 Std:12.98	Mean:31.44 Std:15.54	0.7066
Race	White:34	White:34 Non-White:1	1	White:33	White:33 Non-White:1	1
Sex	M:25 F:9	M:26 F:9	1	M:24 F:9	M:25 F:9	1
pH	Mean:6.60 Std:0.26	Mean:6.47 Std:0.24	*0.03158	Mean:6.61 Std:0.27	Mean:6.47 Std:0.24	*0.0144
Suicide Status	Y:0 N:34	Y:7 N:28	*0.019	Y:0 N:33	Y:7 N:27	*0.019
Smoking at time of death	Y:9 N:8 Unknown:17	Y:23 N:4 Unknown:8	*0.0020	Y:9 N:9 Unknown:15	Y:22 N:4 Unknown:8	*0.0087
Lifetime Alcohol	Heavy in past:2 Heavy in present:0 Little or none:17 Moderate in past:1 Moderate in present:2 Social:12	Heavy in past:3 Heavy in present:9 Little or none:10 Moderate in past:3 Moderate in present:3 Social:7	*0.019	Heavy in past:2 Heavy in present:0 Little or none:18 Moderate in past:1 Moderate in present:2 Social:10	Heavy in past:3 Heavy in present:9 Little or none:10 Moderate in past:2 Moderate in present:3 Social:7	*0.028
Lifetime Drugs	Heavy in past:0 Heavy in present:0 Little or none:29 Moderate in past:0 Moderate in present:1 Social:4 Unknown:0	Heavy in past:3 Heavy in present:6 Little or none:14 Moderate in past:3 Moderate in present:3 Social:4 Unknown:2	*0.0025	Heavy in past:0 Heavy in present:0 Little or none:28 Moderate in past:0 Moderate in present:1 Social:4 Unknown:0	Heavy in past:3 Heavy in present:6 Little or none:14 Moderate in past:3 Moderate in present:2 Social:4 Unknown:2	*0.0042
Anti-psychotics use	Y:0 N:34	Y:35 N:0	*7.27E-16	Y:0 N:33	Y:34 N:0	*2E-15

Results with a significant P-value (≤ 0.05) are identified with a * symbol.

All expression datasets were extracted from *post-morten* prefrontal cortex (BA46A) tissue, using the same biological protocol technique in the same array platform (chip hgu133a—Affymetrix Human Genome U133 Set) comprised by 22,283 genes. Briefly, data was extracted using MAS 5.0 and normalization were performed with R RMA Affy package [23]. Each probe should be considered as detected by MAS 5.0 call (p-value ≥ 0.05) and should be presented in at least 50% of samples in each group. Summarized gene expression was calculated according to median expression of all probes. This totaled in 12,718 (57%) genes expressed. In order to verify if there is a higher variance of gene expression data related to some of the variables (Sex, age, PMI, brain pH and profile), we performed a Single Value Decomposition (SVD) analysis and associated with variables. In BAHN dataset, only Brain pH was observed as significantly associated with three PCs, however the significance of two first PCs ranged from 0.01 up to 0.05. In KATO, age was associated only with second PC. In BAHN dataset, although PC1 and PC2 showed association with Brain pH with a significant p-value, the relationship between them was minimal. In BAHN’s PC1, about 8% of variance found in PC1 can be explained by Brain pH; for PC2, this value was approximately 5%. Regarding the KATO dataset, there was no association found with any sample traits. Considering these results, we opted to not perform any type of adjustment in neither BAHN or KATO datasets.

The methods were applied to each database in an independent way. Results of replication analysis and intersection of the gene lists obtained by both methods are provided for the BAHN and KATO datasets.

### PPI databases and seed genes (GWAS)

In order to perform network analysis using NERI method, three PPI databases presented on Table 2 were merged by union, as done in [13]. Such an operation led to a PPI network with 17,602 nodes (proteins mapped to its original genes, from now on we call PPI nodes as “genes”) and 300,513 edges (interactions). The seed genes list was a manual collection of 38 genes [24] selected based on significant meta-analyses GWAS experiments [25, 26] that presented a extensive evidence with schizophrenia. From 38 *“core genes”*, 30 were successfully mapped to the final PPI network to be considered as seeds (S1 Table). After setting seed genes, the sub-network resulted in 5,212 nodes and 21,142 edges.

**Table 2:** PPI databases adopted for the application of NERI method.

Database	Type of Data	Proteins	Interactions
HPRD	PPI	9,594	38,963
MINT	PPI	5,894	16,934
IntAct	PPI	9,764	41,913
BIOGRID	PPI	16,613	268,351
HPRD ∪ MINT ∪ IntAct ∪ BIOGRID	PPI	17,602	300,513

### WGCNA parameters

We used the parameters suggested in the original documentation of the method [7]. Pearson correlation was used to obtain the correlation matrices needed for the formation of the control and disease groups networks. As it is required that these networks present a scale-free topology, we chose the minimum *β* value which leads to a scale-free independence index (*R*^2^) larger than 0.8. For achieve these result, we removed 2 control samples from BAHN and 6 samples for KATO (5 from control group and 1 from schizophrenia group). The clustering dendogram based on eucledian distance of these samples are avaible on S1 and S2 Figs.

The *β* values obtained for BAHN and KATO were 14 for both datasets. For BAHN’s control and disease networks, the mean connectivity was 4.46 and 4.39, respectively. In relation to KATO’s networks, the mean connectivity for the control network was 5.84 and for the schizophrenia network, 6.24 (the connectivity analysis for all networks are available on S3 and S4 Figs). For all analysis, we used a merge cut high = 0.25, deepsplit = 2.

For the module preservation analysis, we adopted the *Z*_*summary*_ as a measure of preservation a module across two conditions. As it relies on a statistical test based on permutations, the number of permutations was defined as *P* = 100 (default). Finally, the minimum number of genes to form each module was defined as *M* ≥ 50. The genes belonging to the least preserved module are those considered to be potentially associated to the disease (since they are the modules with more topological variation between control and disease groups).

### Module ME correlation with samples traits

To evaluate association of gene co-expressed modules with traits, we used the linear mixed models (LMM) using nlme package of R [27]. Each eingengene module value (kME) were used as y variable in the LMM and the co-variables evaluated were sex, postmortem interval (PMI), age, condition (SCZ or CON) and brain pH. Samples were considered as random effect. ANOVA comparison was used to attributed p-value comparing the null with predicted model. Only associations with ME and traits lower than 0.05 were considered significant.

### NERI parameters

The merged PPI dabatases with the 30 chosen seed genes were used to form two sub-networks (case and control networks) based on the best shortest path for each pair of seeds and each pair of nodes where one is the seed and the other is a neighbor of a seed. The modified version of Kendall’s Concordance Coefficient, as described in [13] was adopted to evaluate the expression concordance of the genes along a given path. The top 10% of the genes presented on the PPI cutting (sub-network resulting from the shortest paths between seeds and its neighbors) were returned according to their Δ′ scores. This led to final lists of 200 for BAHN and KATO, respectively.

### NERI robustness to the chosen seeds analysis

In order to assess the robustness of the NERI method with regard to the seed genes, we performed experiments removing some genes from the original set of 30 seeds but kept the remaining PPI data and expression unchanged. Then, we applied the method taking these altered seed sets as input and analyzed the impact of seed genes subset removal by comparing the resulting lists with the original list. This was performed by removal of multiple seed genes—similar to cross validation. We varied the percentage of excluded seed genes (10%, 20%, 30%, 40%, i.e., removing 3, 6, 9 and 12 seeds, respectively) from the original seeds set. For each percentage of seeds removal, we randomly generated 50 sets of seed genes and applied the NERI method separately for each one. Next, we compared the overlap between the corresponding results with the original resulting list. Then we compared the new gene rankings with the original rankings. For this, we considered only the first elements (top 50, top 100, top 150 and top 200) of each ranking. The rankings were the lists of genes prioritized by Δ′ score.

### Identification of Hub genes in WGCNA and NERI results

For each least conserved WGCNA‘s module, we considered the genes with a kME Pearson correlation ≥ 0.90 as a hub gene. To identify the intra-modular hubs (genes have a high connectivity with other modules) we chose the genes with a ktotal (intra-modular degree) from the 90th to the 99th percentile of all intra-modular connectivity. For NERI results, we considered a hub as a gene with the a degree belonging from the 90th to the 99th percentile of all PPIs (HPRD ∪ MINT ∪ IntAct ∪ BIOGRID) degree.

### Enrichment analysis

For biological function validation of the resulting gene lists, we used the results obtained on MSET analysis. As for the enrichment analysis, DAVID ver. 6.8 (Database for Annotation, Visualization and Integrated Discover) functional annotation were used, for the identification of biological clusters related to cellular components, biological process, molecular functions, diseases and tissue enrichment. For all analysis we choose: Kappa similarity = 3, similarity threshold = 0.5, Initial and Final Group Membership = 3, Multiple Linkage Threshold = 0.5, EASE = 0.1 and Bonferroni score of ≤ 0.05. The networks based on DAVID results were made using the software Gephi 0.9.2 [28] and we used the ClusterProfiler R package [29] to create the KEGG‘s enrichment pathways results and plots.

### MSET analysis

For the background of MSET analysis [20], we used all 12,719 genes (as seems on 1 (WGCNA), and the 5,108 genes (S4 Table) used on NERI PPI network. For the reference list, we chose 5 databases related to schizophrenia available on SZDB (http://www.szdb.org/), based respectively on CNV (28 genes), exome (613 transcripts), 152 differentially methylated genes, 1,817 differentially expressed genes and GWAS (484 genes) experiments S2 Table. All tests adopted 10,000 permutation per analysis.

## Results

### WGCNA’s least preserved module and NERI’s Δ′ score genes analysis

The WGCNA least preserved module represents a final network of 239 (BAHN) and 365 genes (KATO), as seem on S1 and S2 Figs and S3 Table. In both images, the x axis represents the module size (or the number of genes of each module) and y axis shows the preservation Zsummary score. For BAHN database, excluding the grey (null module), 24 modules were formed—with the RoyalBlue module achieving the lowest preservation according to both the medianrank (24) and Zsummary (10) scores (S1 Fig). KATO dataset resulted in 15 modules with the Greenyellow with the lowest preservation Score medianrank (15.5) and Zsummary (7) scores (S2 Fig). By using NERI, in its turn, the final gene list obtained by the Δ′ score was 200 genes for both BAHN and KATO databases as seem on S3 Table.

### WGCNA: Module ME correlation with samples traits

**Fig 2:**
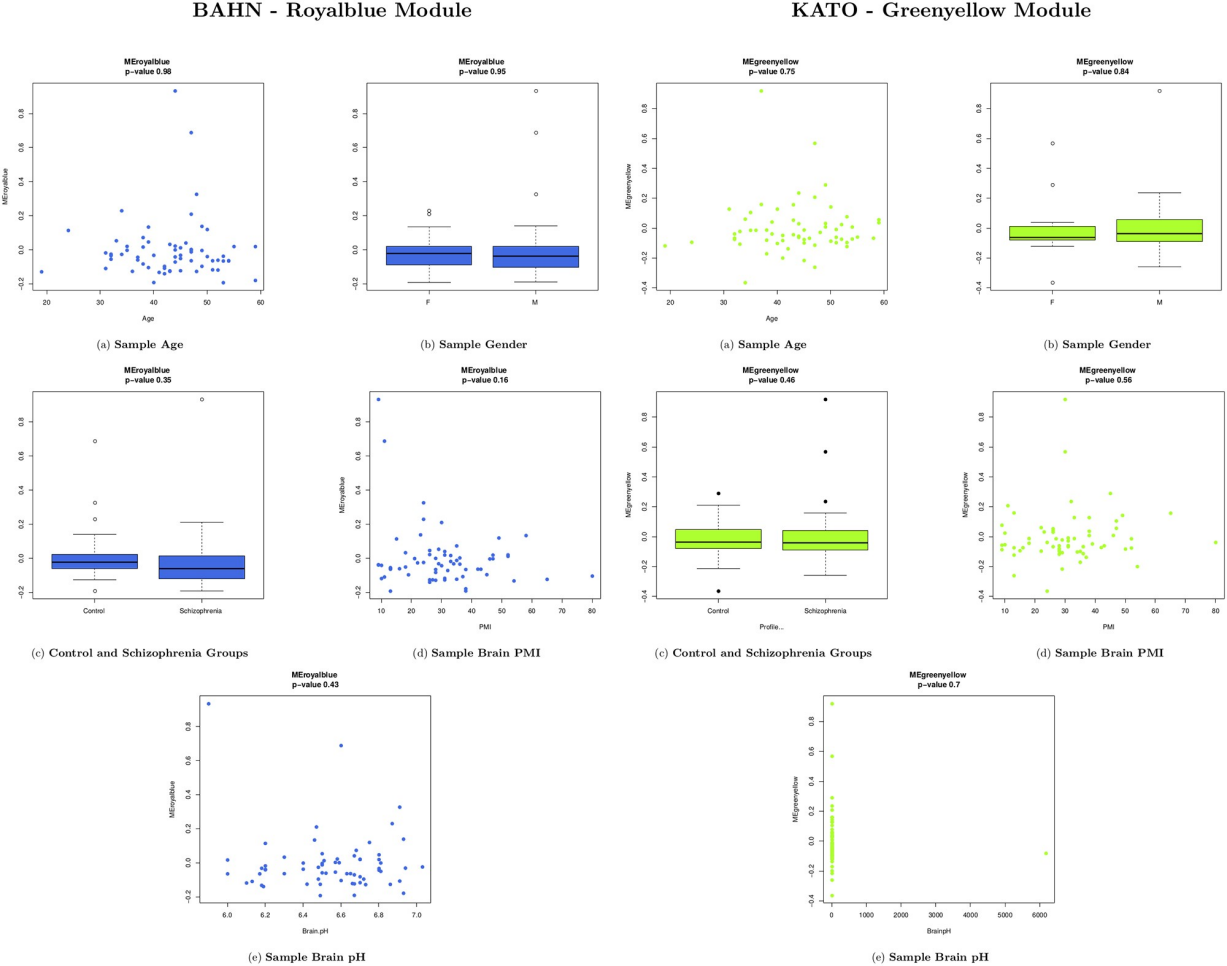
Correlation between BAHN and KATO least preserved module (Royalblue and Greenyellow, respectively) kME and sample traits, using LME model. In both results, Age (a), Gender (b), Disease (c), PMI (d) and brain pH (e). In all subfigures, the kME M value are inserted on y-axis and the sample trait, on x-axis.

According to the LME model (Fig 2), For BAHN dataset modules purple had association with sex (p = 0.036) and ligthgreen with Schizophrenia (p = 0.038)—S6 Fig. The least preserved module Royalblue ME value presented no association with age (p = 0.98), sex (p = 0.95), brain pH (p = 0.43), schizophrenia (p = 0.25) or post-morten interval (p = 0.36). KATO dataset modules only tan (p = 0.0062) and red (p = 0.0076) modules presented association with sex (S7 Fig), with the Greenyellow as the least preserved module. The Greenyellow ME value presented no association with Age (p = 0.75), sex (p = 0.84), Schizophrenia (p = 0.72), post-mortem interval (p = 0.56) and brain pH (p = 0.70).

### NERI robustness analysis

**Fig 3:**
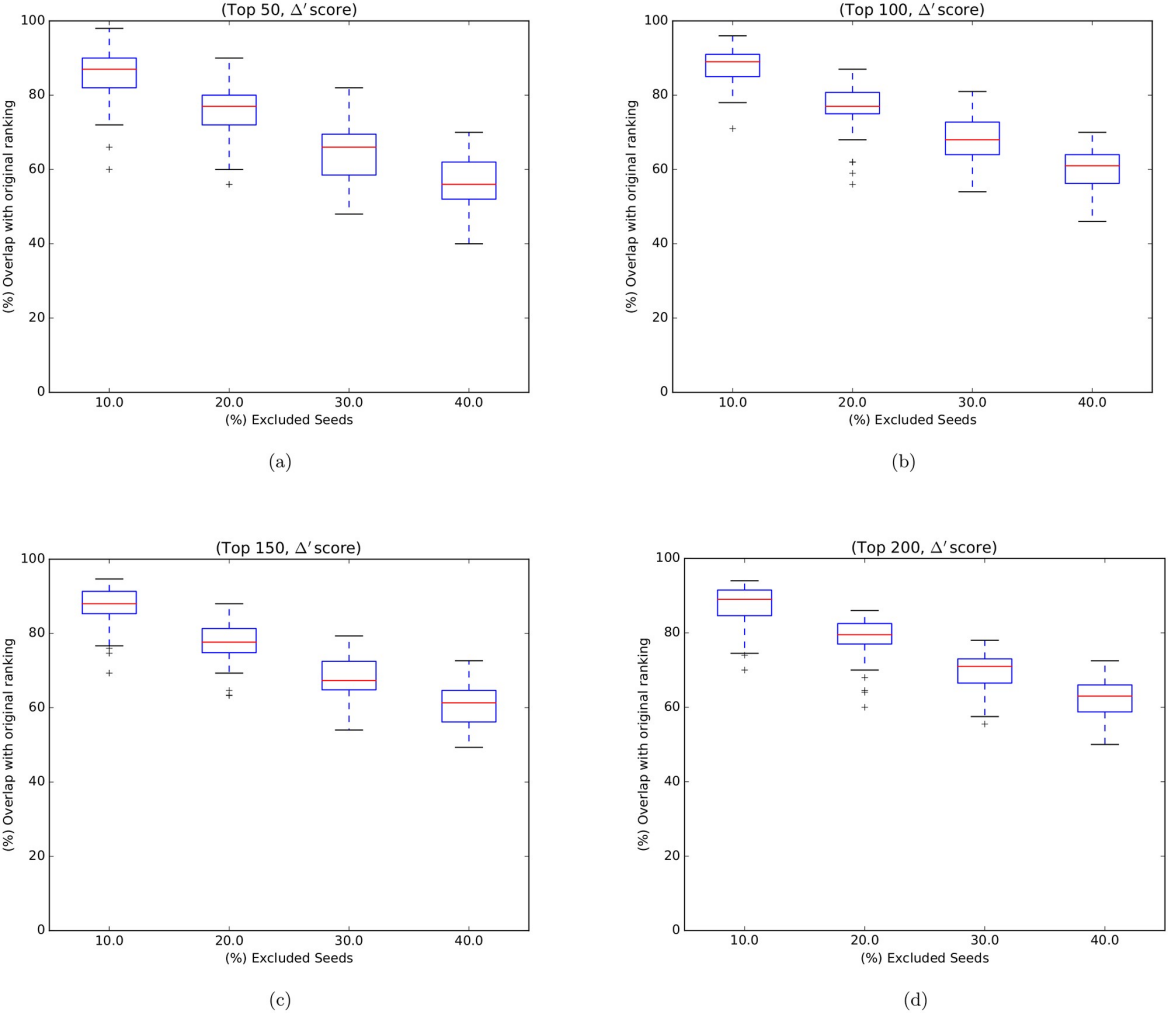
Boxplots representing NERI robustness overlap distributions: Boxplots representing the distributions of 50 overlaps (corresponding to 50 random seed sets, one for each execution) for the top 50, 100, 150, 200 genes ranked by Δ’ score. The X axis represents the proportion of removed seeds (10%, 20%, 30%, 40%, which correspond to 3, 6, 9, 12 removed seeds respectively). The Y axis represents the overlaps distributions in percentage.

Fig 3 shows boxplots representing the distributions of 50 overlaps (corresponding to 50 random seed sets, one for each execution) for the top 50, 100, 150, 200 genes ranked by Δ’ score. We note that the higher the number (3, 6, 9 and 12) of seeds removed, the greater the impact on the rankings, as expected. Given the above findings, we conclude that the method is relatively robust to moderate variations in the seeds list.

### Replication analysis

**Fig 4:**
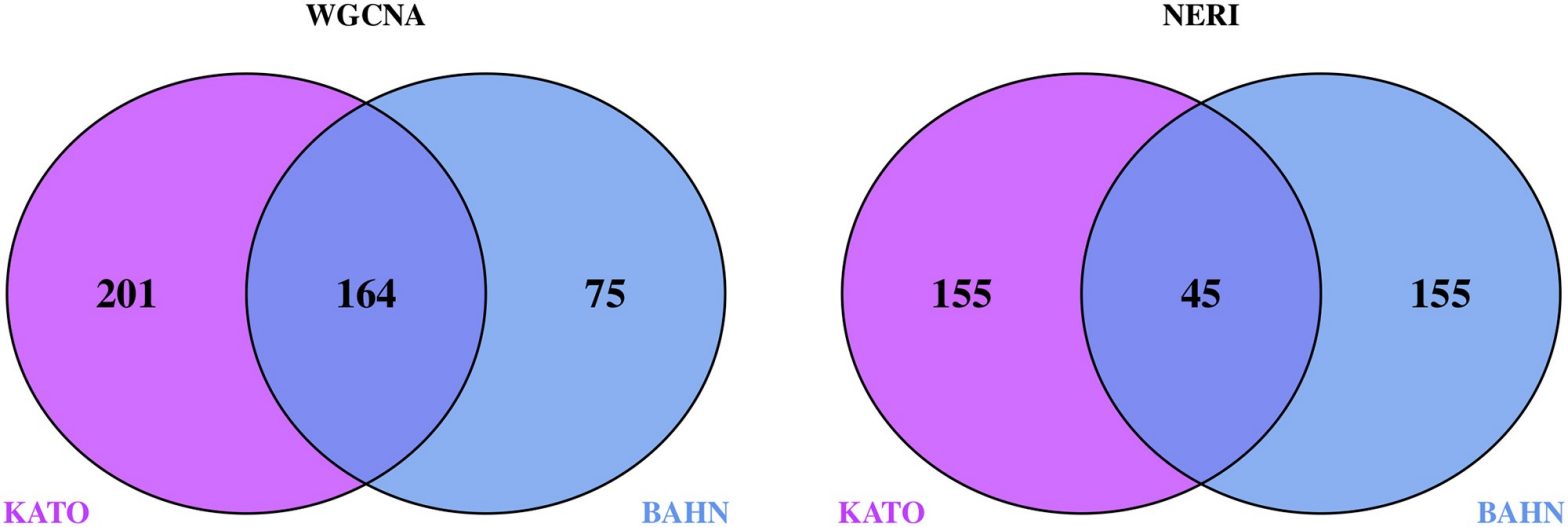
WGCNA—Least preserved modules (A) and NERI—Δ′ SCORE (B) Replication analysis of the intersection obtained from the final results obtained by WGCNA and NERI for the 2 databases: BAHN and KATO. (A) WGCNA, least preserved module (total N = 12,719 genes); (B) NERI, Δ′ score (total N = 9,554 genes).

**Fig 5:**
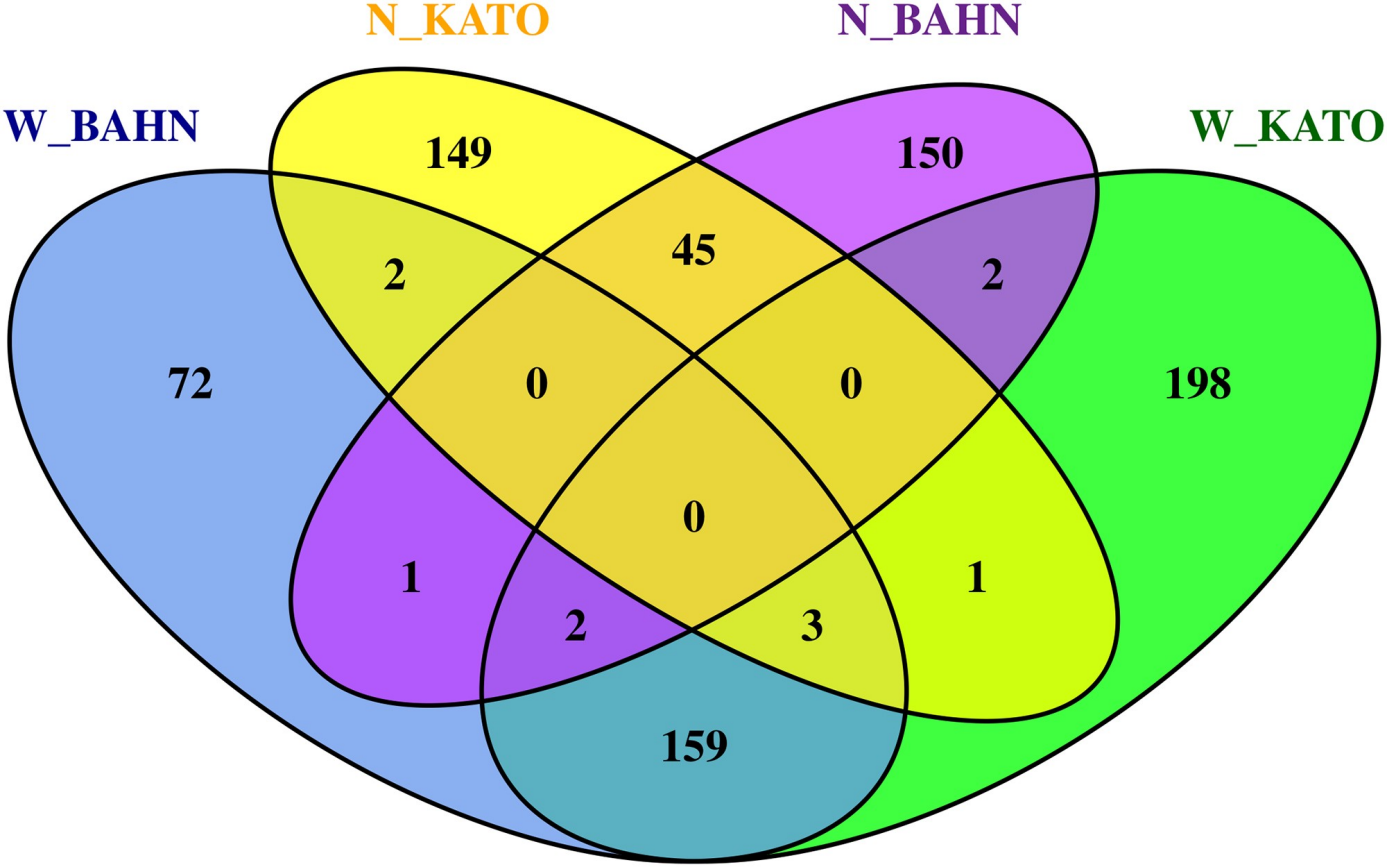
Final intersection analysis of BAHN and KATO—WGCNA x NERI intersections between BAHN and KATO dabatase on each one of WGCNA and NERI results presented on the venn diagrams on Fig 4. Results with W_ pattern represents the WGCNA’s least preserved module; N_ represents the genes from NERI‘s Delta ranking.

In order to compare the final results produced by the two methodologies, we performed a replication analysis using the results of the 2 different microarray datasets (BAHN and KATO). Figs 4 and 5 indicates the intersection between both WGCNA and NERI across BAHN and KATO databases.

### Enrichment results

**Fig 6:**
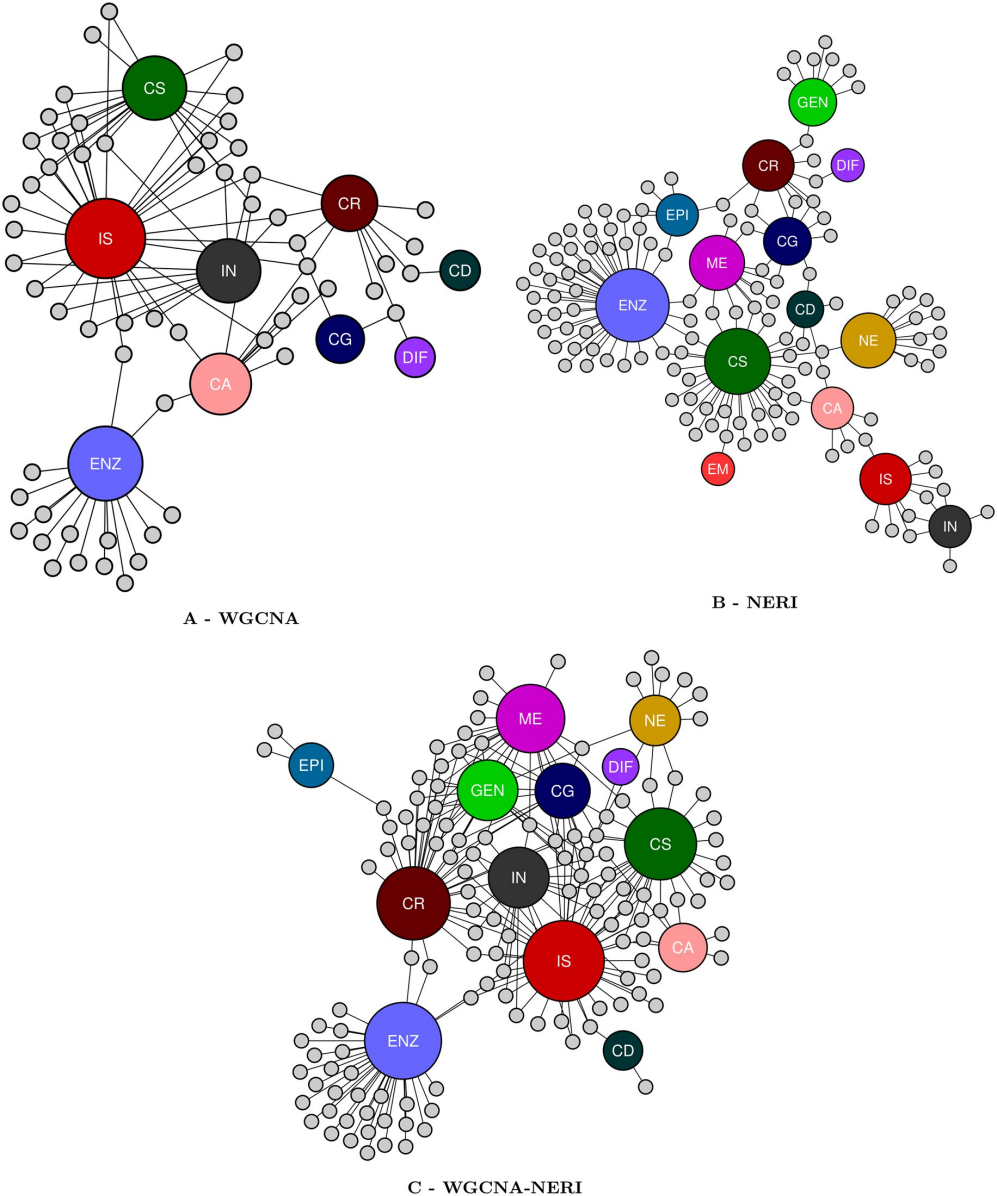
DAVID clusters based on its biological categories of WGCNA, NERI and WGCNA-NERI results from BAHN database. The network based on DAVID results of WGCNA-NERI, as seem on Table 3, from BAHN database. On its center (small nodes) all identified clusters; on the outer networks, represented by larger nodes, each one of the 11 biological categories, as identified on Table 3.

**Fig 7:**
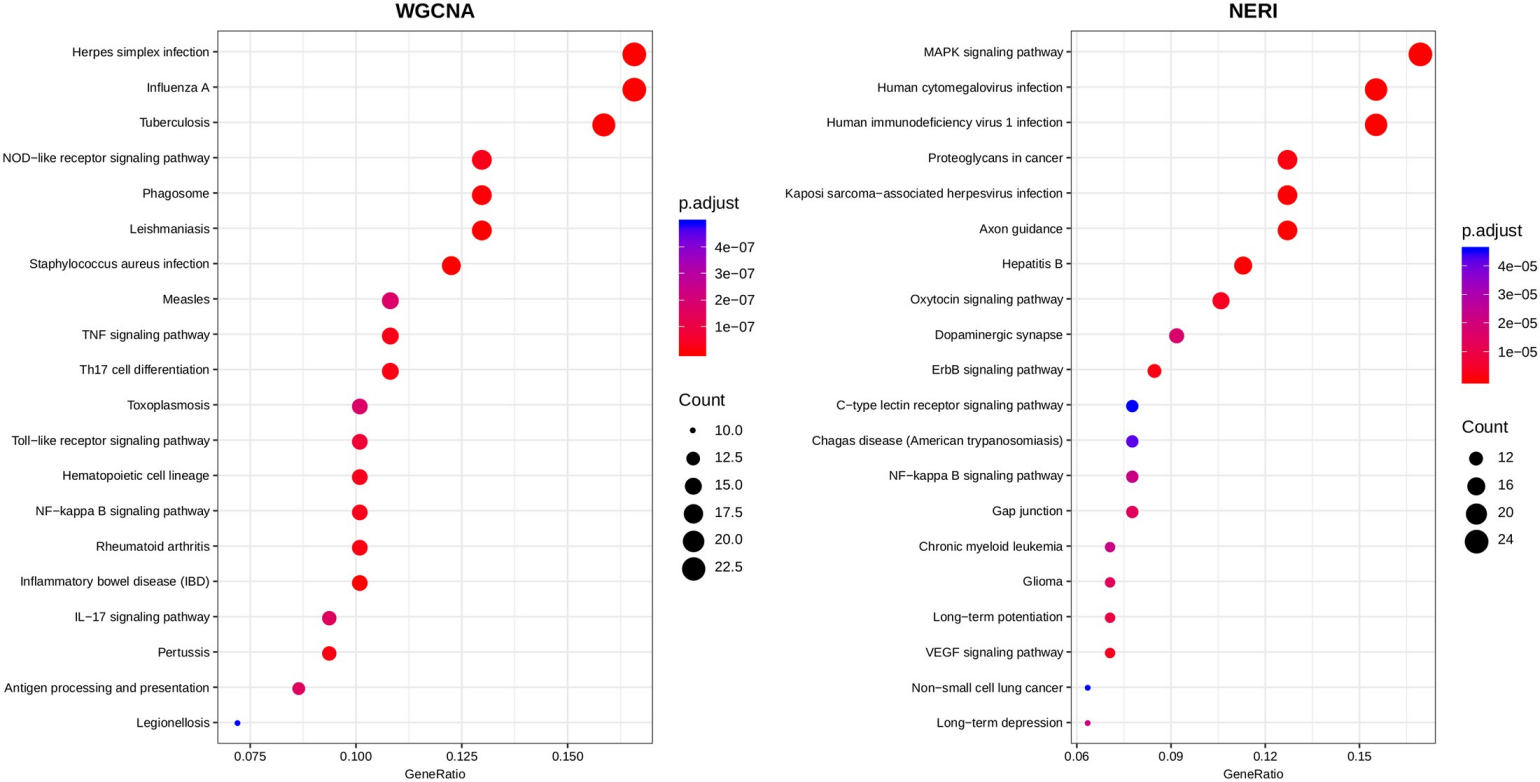
The TOP 20 pathways (ranked by FDR ≤ 0.05) from KEGG database using the BAHN dataset. In both plots, the intersection between the list of genes obtained by both WGCNA least preserved module (right) and NERI (left). The size of each circles represents the counts (number of genes that are part of each pathway). The pathways are listed on y-axis and on the x-axis, the Gene Ratio of each result.

All results obtained on DAVID are on S5 Table. Table 3 summarizes the clusters obtained in all 6 analysis. These clusters contained a different number of pathways, ranging from cellular regulation, cellular signaling, immune response, inflammation, metabolic pathways, cellular differentiation, genetic and epigenetic pathways, cellular growth, nervous system activity, embryonic activity and general cellular activity. We only considerate the clusters with contained pathways a Bonferroni score of ≤ 0.05 (The CLS column in this table indicates the total number of clusters formed by DAVID per analysis). Fig 6 illustrates only the results from BAHN database—each of these biological categories are represented by the different colors of the larger nodes; the smaller nodes with a grey color represents each one of the clusters with a Bonferroni score of ≤ 0.05, with it’s edges presents the biological categories of the pathways of each cluster. Fig 7 represents the enrichments of the TOP 20 pathways (ranked by FDR ≤ 0.05) for KEGG database. The complete list are enriched pathways are available on S6 Table.

**Table 3:** The classification of the biological pathways (Gene Ontology) of each cluster obtained on DAVID, accordingly with WGCNA, NERI and WGCNA-NERI results. DB = database, CA = Cellular Activity, CD = All types of cellular death or apoptosis, CG = Cellular Growth, CR = cellular regulation, CS = cellular signaling, DIF = cellular differentiation, ENZ = protein activity, GEN = genetic related functions, IN = inflammatory pathways, IS = immune system, ME = metabolism, NE = nervous system, EM = embryological activity, CL = Total number of formed Clusters on each analysis, filtered by Bonferroni ≤ 0.05. The complete list of the enriched pathways are available on S5 Table.

Method	DB	CA	CD	CG	CR	CS	DIF	EM	ENZ	EPI	GEN	IN	IS	ME	NE	CL
WGCNA	BAHN	9	1	2	10	19	1	0	16	0	0	10	30	0	0	34
	KATO	4	6	0	0	14	0	1	13	0	1	11	31	0	0	43
NERI	BAHN	6	5	8	9	32	1	1	44	6	8	6	9	11	11	34
	KATO	4	2	12	8	17	0	0	37	4	2	3	9	6	7	55
WGCNA-NERI	BAHN	7	2	13	33	26	1	0	34	3	16	16	41	22	10	57
	KATO	3	2	5	13	20	2	5	12	0	3	6	18	2	0	42

### MSET analysis

The Table 4 indicates the MSET analysis based on NERI, WGCNA and our novel approach, WGCNA-NERI. Both WGCNA and NERI algorithms presented a significant p-value (≤ 0.05) for the diferentially expressed genes data, with respectively 40 and 28 genes (for BAHN dataset) and for KATO dataset, 52 and 32 genes. Our approach, WGCNA-NERI also achieved a significant p-value in both datasets, increasing the number of genes for both datasets to 66 and 82 differentialy expressed genes according to the SZDB database. Despite none of the others datasets (CNV, DMG, exome and GWAS) achieved a significant p-value, is possible to observe that in all cases (with the exception of CNV—BAHN), the WGCNA-NERI approach increased the number of the overlap—when comparing with the WGCNA and NERI results in separate.

**Table 4:** MSET analysis of the intersection of BAHN and KATO results (of NERI, WGCNA and WGCNA-NERI methods) with reference the sets for Copy Number Variant (CNV), Differentially Expressed Genes (DEG), Differentially Methylated Genes (DMG), Exome (EXOM), Genome Wide Association Study (GWAS). All reference sets provided by SZDB (A database for schizophrenia genetic research).

Method	Dataset	CNV / #	DEG / #	DMG / #	EXOM / #	GWAS / #
WGCNA	BAHN	1 (0)	< 0.001 (40)*	0.32 (3)	0.70 (7)	0.34 (7)
	KATO	0.48 (2)	< 0.001 (52)*	0.36(4)	0.70 (11)	0.97 (4)
NERI	BAHN	0.9 (2)	0.04 (28)*	0.61 (2)	0.68 (8)	0.92 (3)
	KATO	0.09 (2)	0.005 (32)*	0.34 (3)	0.41 (10)	0.50 (6)
WGCNA-NERI	BAHN	0.1 (2)	< 0.0001 (66)*	0.31 (5)	0.50 (15)	0.60 (10)
	KATO	0.07 (3)	< 0.0001 (82)*	0.18 (7)	0.34 (21)	0.86 (10)

Results with a significant P-value (≤ 0.05) are identified with a * symbol.

### Identification of hub genes in WGCNA‘s least preserved module and NERI‘s Δ′ score

In the WGCNA analysis (BAHN dataset), we identified 18 hubs genes contained in Royalblue Module (n = 239) as well as 4 intra-hub genes. In relation to the KATO dataset, 33 hubs genes were identified on Greenyellow Module (n = 365), with 2 intra-hub genes. For NERI‘s top 200 genes for both BAHN and KATO dataset respectively, we identified 75 and 66 hub-genes. Comparing the list of hub genes from both WGCNA and NERI, there were no overlap between the hub genes of both methods.

To evaluate whether the schizophrenia’s differentially expressed genes (SZDB) obtained on the MSET analysis were in fact, hub genes in WGCNA-NERI results, we compared the overlap between these genes. From the 66 DEG obtained on BAHN results, 37 were considered hub genes. For KATO results (82 genes), we obtained an overlap of 19 hub genes from both WGCNA and NERI’s hubs. The list of hub genes of each result, as well it‘s overlap between the SZDB DEG list are avaible on S7 Table.

In order to compare the final results produced by the two methodologies, we performed a replication analysis using the results of the 2 different microarray datasets (BAHN and KATO). Figs 4 and 5 indicates the intersection between both WGCNA and NERI across BAHN and KATO databases.

## Discussion

WGCNA results provided a network of 239 genes (BAHN) and 365 genes (KATO), while NERI Δ′ resulted on 200 genes in both databases. In WGCNA analysis, we opted to choose the least preserved module according to medianRank summary, as in both datasets the Z summary score was high. None of the least preserved modules were associated with variables used (including disease status), it doesn’t discard the possibility of association with disease. The only module that was associated with disease status was the Lightgreen in BAHN dataset. This module presented a lncRNA *MEG3*, which is an imprinted gene. Its role in long-term potentiation (LTP) was observed in primary cortical neurons [30], and altered expression of these gene was already related to drug abusers [31]. In NERI analysis, we first tested for its robustness regarding seed genes. Even the change of up to 40% of initial seeds, the overlapping of final genes was high ranging 50–60%. This showed that due to methodological approach used by NERI algorithm the results are similar. Accordingly to the replication analysis, both results obtained a significant overlap (164 genes for WGCNA and 45 to NERI). NERI are a suited method for replication across different databases [32], with can be expected (since it’s core method utilizes PPI databases for it’s network analysis based on shortest path ranking and WGCNA is completely dependent only on gene expression). Considering that both datasets was extracted from the same individuals, is unknown if the overlap of the WGCNA would remain relevant with across different experiments.

Differences regarding both methodical approaches are: NERI works only with protein coding gene, and its PPIs are based in the literature knowledge, the searching for biological functional pathways is limited to PPI dimensional space. On the other hand WGCNA works with co-expression matrix which can carry a dataset bias, however the gene module co-expression construction can be performed based on the pattern of expression data with no previous knowledge in the literature. Moreover, as it works at transcriptional levels several lncRNAs could be addressed, which could present important roles to disease/disorder, as observed by *MEG3* in Lightgreen module.

To evaluate the biological function of the selected genes and in order to investigate whether we could observe new functional categories enriched using our approach (WGCNA-NERI), we also explored enrichment in the functional categories using DAVID ver.6.8. The results were summarized on Table 3. It’s possible to observe the difference between the number of the clusters related to each biological pathways across each method. In WGCNA results from both BAHN and KATO, most clusters related to immune system (IS) activity, inflammation (IN), cell signaling pathways (CS), and cellular regulation process (CR). In other way, the NERI highlighted clusters involved to metabolic pathways (ME), cellular regulation, cellular activity (CA), protein activity (ENZ), cellular growth (CG), genetic (GEN) and epigenetic (EPI) pathways and also a larger number of central nervous system activity (such as axonal growth, neurogenesis) when compared to WGCNA clusters. It’s possible to observe the tendency of WGCNA to provide clusters related to immune system activity, inflammatory pathways and cellular regulation. NERI provides different results, more related to cellular regulation, nervous system, embryological pathways and also cellular activity and metabolism.

Based on DAVID’s enrichment analysis, it’s possible to infer that our approach, WGCNA-NERI, was capable to summarize both methods results, suggesting a promising approach to study a complex psychiatric disease.

Also, it’s possible to observe on Table 3 that some GO categories were less represented in WGCNA-NERI lists than each method in separate—as for example, Nervous System (NE) pathways, which on NERI alone, presented 11 and 7 clusters and on WGCNA-NERI, 10 and 0 clusters. This could be a bias derived from the short number of components of the enriched NE pathways (which tends to be small when compared to cell signaling or cellular regulation pathways, for example). As WGCNA-NERI approach uses a larger list than each method in separate, this could lead to a bias in the enrichment analysis of pathways with less components.

We used each of WGCNA and NERI results (and our new approach, WGCNA-NERI, which consists of the combination of WGCNA’s least preserved module and NERI’s Δ′ score) and set the MSET analysis using 5 databases related to CNV, GWAS, Exome, DEG and DMG. None of the analysis were able to achieve a relevant *pvalue* related to the CNV, GWAS, Exome and DMG sets—this was possible only with the DEG datasets. Probably this results reflect the fact that both methods are based on gene expression data using network approaches to select genes that are altered in the disorder. However, is possible to observe that in almost all cases (with the exception of WGCNA-NERI BAHN CNV), the WGCNA-NERI approach provided a increase in the number of overlap genes of each set—which could be a evidence that both algorithms identify different aspects (or biological pathways) related to the schizophrenia.

In relation to the genes belonging to the schizophrenia DEG of SZDB database, the WGCNA-NERI approach produced a overlap of 66 genes for BAHN dataset and 82 genes for the KATO dataset. Additionally, there were an overlap of 36 genes (or 32.1%) between both results. For the 30 exclusive DEG for BAHN overlap, AQP1 (Aquaporin 1 Colton Blood Group) is a water channel molecule that is expressed in the central nervous system and was previously related to neurodegenerative and neurological disorders [33, 34]. JMJD6 (jumonji domain containing 6) was found as one of the regulators of embryological organogenesis in mouses and as well into general brain development [35]. The SLC6A3 gene (solute carrier family 6 member 3) encodes a dopamine transporter (DAT) related to the active re-uptake of dopamine neurotransmisser—and as such, is related to some neurogenetive disorders (such as Parksons disease), neurodevelopmental disorders and memory [36–38].

The 46 unique DEG related to KATO dataset, BCL6 gene (B cell CLL/lymphoma 6) is involved in neurogenesis, mainly in the differentiation or cerebellar granule cells and general cerebellar development [39, 40]. DIAPH3 (diaphanous related formin 3) is a gene involved in cell movement and adhesion, and may be involved in autism spectrum disorders [41], as well to microcephaly [42]. JAK2 (Janus kinase 2) is part of the JAK/STAT signaling pathway and is also involved on the regulation of glutamate transporters [43, 44].

The overlap between SZGB DEG from both BAHN and KATO datasets (36 genes) presented genes such as A2M (Alpha2-macroglobulin), an neuroinflamatory biomarker [45] and COL4A1 (Collagen type IV alpha 1 chain), particularly related the vascular system of the cerebrum [46].

Using the overlap between SZGB’s DEG and WGCNA-NERI hub genes (37 hub genes for BAHN and 19 hub genes for KATO), we identified genes related to neuroimmunity and neuroinflammation such as as CHI3L1 (Chitinase 3 Like 1) and CHI3L2 (Chitinase 3 Like 2), both genes related to the degeneration of motor neurons in amyotrophic lateral sclerosis (ALS) as well to neuroinflammatory processes in general [47–49]; IL6 (Interleukin 6), a mediator of TH2 (T helpers cells), involved as well into embryogenesis [50]; CXCL9 (C-X-C Motif Chemokine Ligand 9), protein coding gene related to T cell trafficking [51].

Genes related to synaptic transmission and schizophrenia were hubs in WGCNA-NERI resuls: EEF2 gene (Eukaryotic Translation Elongation Factor 2) encodes a GTP-binding translation elongation factor family, part of the eEF2K/eEF2 pathway, regulating the GABAergic and serotonergic (5-HT) synapses [52, 53]; SERPINA 3 (Serpin Family A Member 3), a plasma protease inhibitor produced also by the astrocytes, was previously related to the neuroinflammation aspect of the schizophrenia and other psychiatric and neurodegenerative disorders [53–55]; PRKCA (Protein Kinase C Alpha), related to the memory impairment in schizophrenia as well with bipolar disorder [56–58].

Most importantly, our approach was able to identify more genes previously related to schizophrenia disorder, identifying all of these genes (whereas each method by itself could not point out). This suggest that the set of genes identified using both methods could also be important in the context of this disorder. Also, Table 4, shows that not the only p-value performed better using our WGCNA-NERI approach, but it was also able to identify more genes previously reported in the literature than each method by itself. As predicted, the lists obtained by WGCNA and NERI lead to a set of genes with different biological functions, but a expressive quantity of these are components of the same schizophrenia genomic network (as indicate on transcriptome, methylation and *de novo* variations of SZDB database).

## Conclusion

Our proposal was to evaluate the complementarity between the results of WGCNA’s least preserved modules and NERI Δ′ score, using schizophrenia as a case study and propose a new approach, using the results from both methods. WGCNA and NERI assumes parts of the Network Medicine hypotheses to derive potentially relevant networks from the biological point of view. Both methods not only derive two networks, one for control condition and another for disease condition, but also perform differential analysis between these two networks. While WGCNA ranks modules of genes according to their topological preservation across control and disease co-expression networks, NERI ranks genes according to their relative importances in both control and disease networks extracted from the integration of PPI networks, gene expression data and seed genes previously known to be associated with the disease (for example, by genome-wide association studies).

By retrieving genes from the least preserved modules obtained by WGCNA and from top 10% NERI Δ′ scores (based on largest sum of relative importances in both conditions), when applied to two different gene expression databases containing control and schizophrenia samples, both methods displayed genes directly related to different biological pathways of schizophrenia. For instance, the WGCNA results were more associated with inflammatory pathways and immune system response; NERI obtained genes related with cellular regulation, embryological pathways e cellular growth factors.

Both methods performed well on replication analysis—it’s unknown if the WGCNA would be capable to offer similar results when using data from different types of biological tissue (BAHN and KATO database experiments were made with the same biological samples). Both algorithms provided a statistical meaningful result to the MSET analysis using genes provided by the SZGB database (only for the DEG dataset). Combining WGCNA and NERI provided a much more larger overlap in this category—as well to the other datasets, altought with a p-value ≥ 0.05. Moreover, the increased overlap in all categories obtained by WGCNA-NERI approach could be an indication that both algorithms are in fact, prioritizing different genes directly related to the schizophrenia, regarding the DEG, CNV, exome, DMG and GWAS data.

Our new approach, WGCNA-NERI was capable to combine both results, in that way, our study suggests that using both methods in combination is better for establishing a group of modules and pathways related to a complex disease than using each method individually.

## Supporting information

S1 Fig WGCNA—Samples clustering, kME royalblue module correlation and module preservation statistics (BAHN Dataset). On (a): clustering dendogram based on euclidian distance (y axis) of the control and schizophrenia samples; (b): Royalblue kME pearson correlation with the expression values of all 239 genes contained on Royalblue Module control group (x axis) and schizophrenia group (y axis) with corp = 0.68. The genes labeled with the red color represents the genes with the larger ratio of pearson‘s correlation between control and schizophrenia groups; (c): Module Preservation Statistics. The Royalblue module was the least preserved according to both Median Rank and Zsummary metrics.
(TIFF)

S2 Fig WGCNA—Samples clustering, kME greenyellow module correlation and module preservation statistics (KATO Dataset). On (a): clustering dendogram based on euclidian distance (y axis) of the control and schizophrenia samples; (b): Greenyellow kME pearson correlation with the expression values of all 365 genes contained on Royalblue Module control group (x axis) and schizophrenia group (y axis) with corp = 0.5. The genes labeled with the red color represents the genes with the larger ratio of pearson‘s correlation between control and schizophrenia groups; (c): Module Preservation Statistics. The Greenyellow module was the least preserved according to both Median Rank and Zsummary metrics.
(TIFF)

S3 Fig WGCNA—Connectivity analysis from control and schizophrenia networks (BAHN dataset), representing the scale free topology model fit, mean, maximum and median connectivity. In all subfigures, each number represents a different power (or *β* value) for both control (blue color) and schizophrenia (red color) networks. For both networks, the *β* value chosen was 14.
(TIFF)

S4 Fig WGCNA—Connectivity analysis from control and schizophrenia networks (KATO dataset), representing the scale free topology model fit, mean, maximum and median connectivity. In all subfigures, each number represents a different power (or *β* value) for both control (blue color) and schizophrenia (red color) networks. For both networks, the *β* value chosen was 14.
(TIFF)

S5 Fig WGCNA—Singular value decomposition analysis (SVD). On (a): clustering dendogram based on euclidian distance (y axis) of the control and schizophrenia samples; (b): Greenyellow kME pearson correlation with the expression values of all 365 genes contained on Royalblue Module control group (x axis) and schizophrenia group (y axis) with corp = 0.5.
(TIFF)

S6 Fig WGCNA—Module kME correlation with sample traits (BAHN). Age, Brain pH, PMI, Disease status (Profile) and Gender (sex) are represented on the y-axis. The p-values are represented on a -log10 scale.
(TIFF)

S7 Fig WGCNA—Module kME correlation with sample traits (KATO). Age, Brain pH, PMI, Disease status (Profile) and Gender (sex) are represented on the y-axis. The p-values are represented on a -log10 scale.
(TIFF)

S1 Table List of the 37 “core genes” related to schizophreniaused in NERI method (based on Jia et al. 2011). (XLSX)

S2 Table List of genes related to the SZDB database used on MSET analysis. (XLSX)

S3 Table List of genes obtained by WGCNA least preserved module, NERI TOP 200 genes ranked by Delta’ and WGCNA-NERI—related to BAHN and KATO expression data. (XLSX)

S4 Table Complete list of NERI Delta’ score obtained by BAHN and KATO expression data. (XLSX)

S5 Table Complete list of DAVID (ver.6.8) enrichment clusters. (XLSX)

S6 Table Complete list of KEGG enrichment results. (XLSX)

S7 Table List of hub genes obtained on both WGCNA’s least preserved module and NERI’s Delta’ score. (XLSX)
