# Supplementary figures and images for: Assessment of complementarity of WGCNA and NERI results for identification of modules associated to schizophrenia spectrum disorders
Source: PLoS One. 2019 Jan 15;14(1):e0210431. doi: 10.1371/journal.pone.0210431 (PMC6333352; doi:10.1371/journal.pone.0210431)

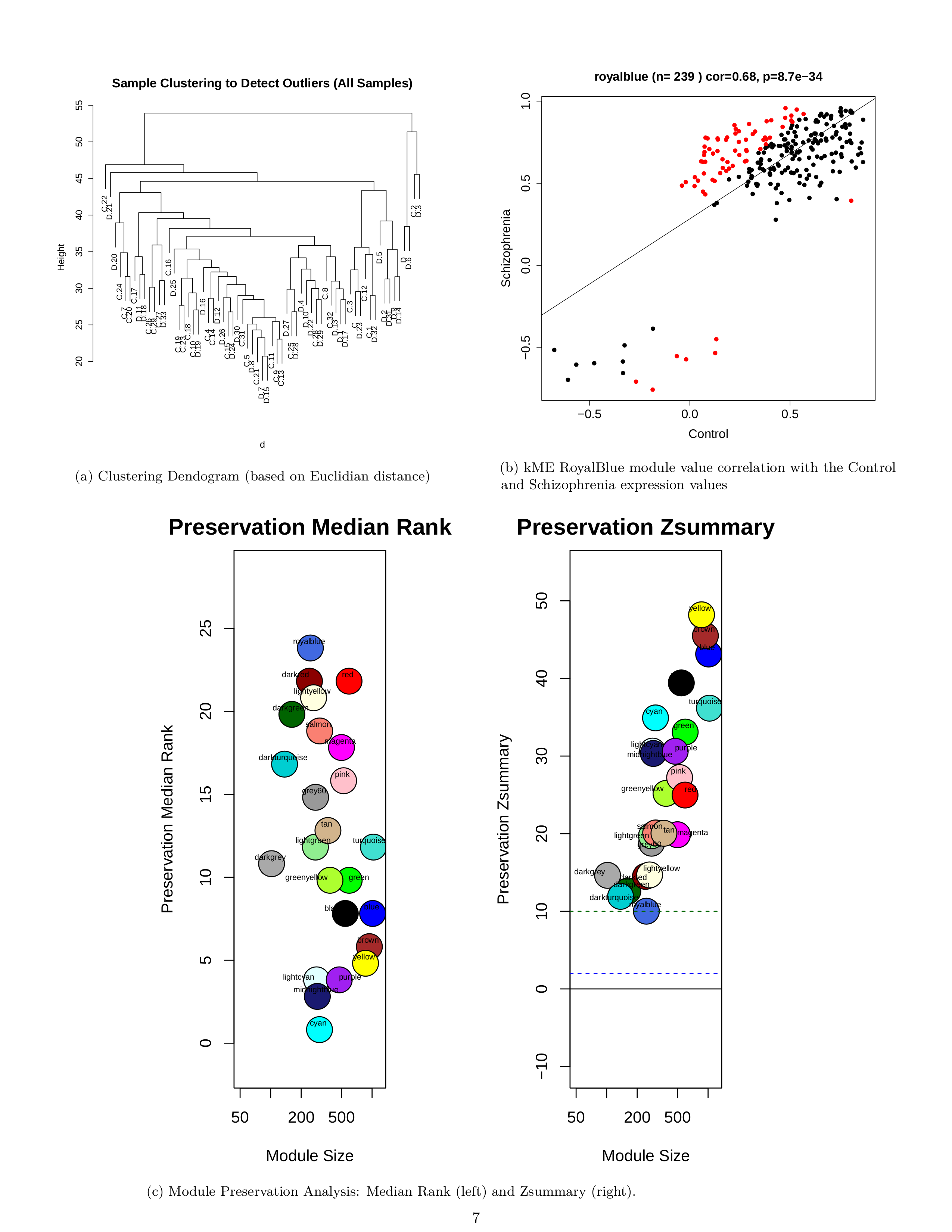

Supplement: S1 Fig — On (a): clustering dendogram based on euclidian distance (y axis) of the control and schizophrenia samples; (b): Royalblue kME pearson correlation with the expression values of all 239 genes contained on Royalblue Module control group (x axis) and schizophrenia group (y axis) with corp = 0.68. The genes labeled with the red color represents the genes with the larger ratio of pearson‘s correlation between control and schizophrenia groups; (c): Module Preservation Statistics. The Royalblue module was the least preserved according to both Median Rank and Zsummary metrics. (TIFF) [file pone.0210431.s001.tiff]

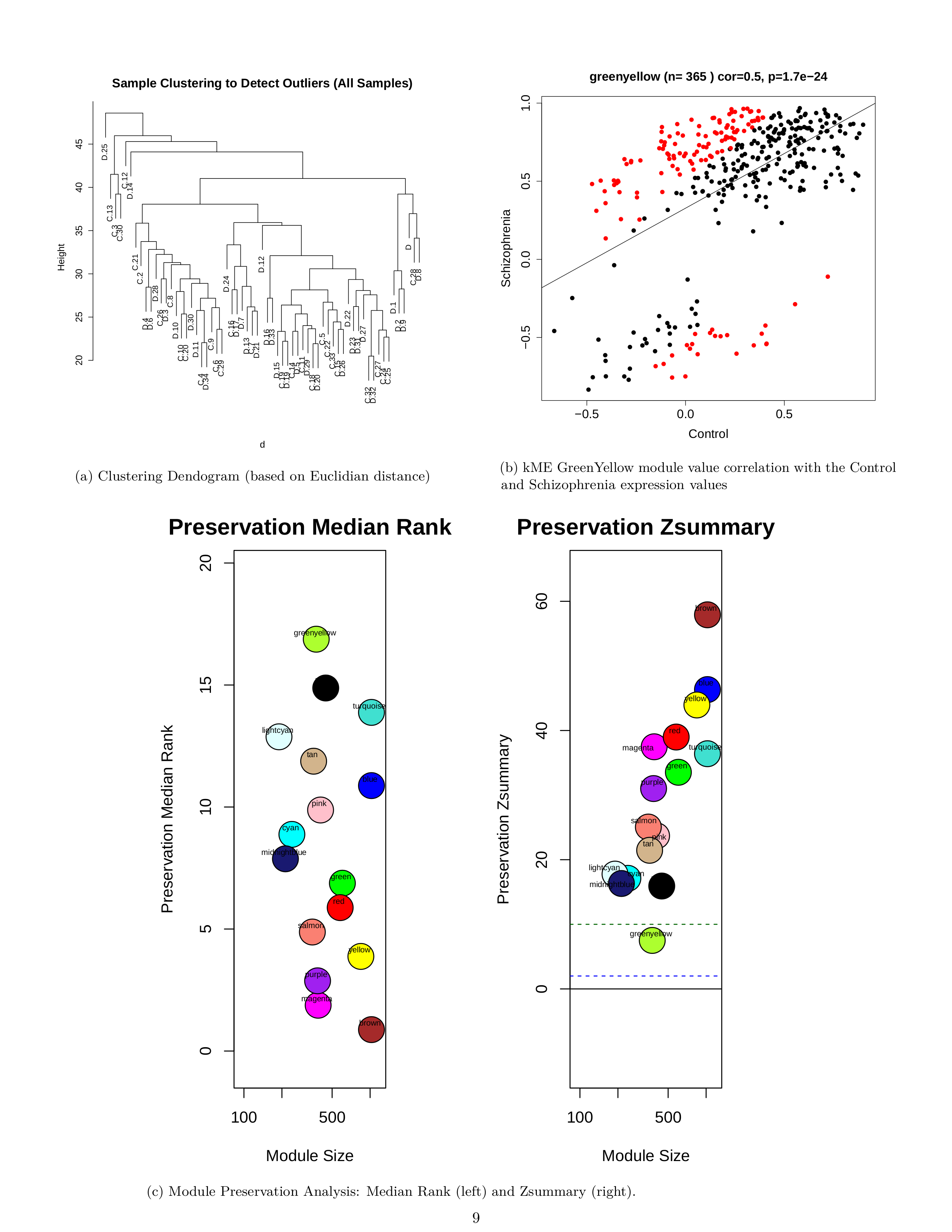

Supplement: S2 Fig — On (a): clustering dendogram based on euclidian distance (y axis) of the control and schizophrenia samples; (b): Greenyellow kME pearson correlation with the expression values of all 365 genes contained on Royalblue Module control group (x axis) and schizophrenia group (y axis) with corp = 0.5. The genes labeled with the red color represents the genes with the larger ratio of pearson‘s correlation between control and schizophrenia groups; (c): Module Preservation Statistics. The Greenyellow module was the least preserved according to both Median Rank and Zsummary metrics. (TIFF) [file pone.0210431.s002.tiff]

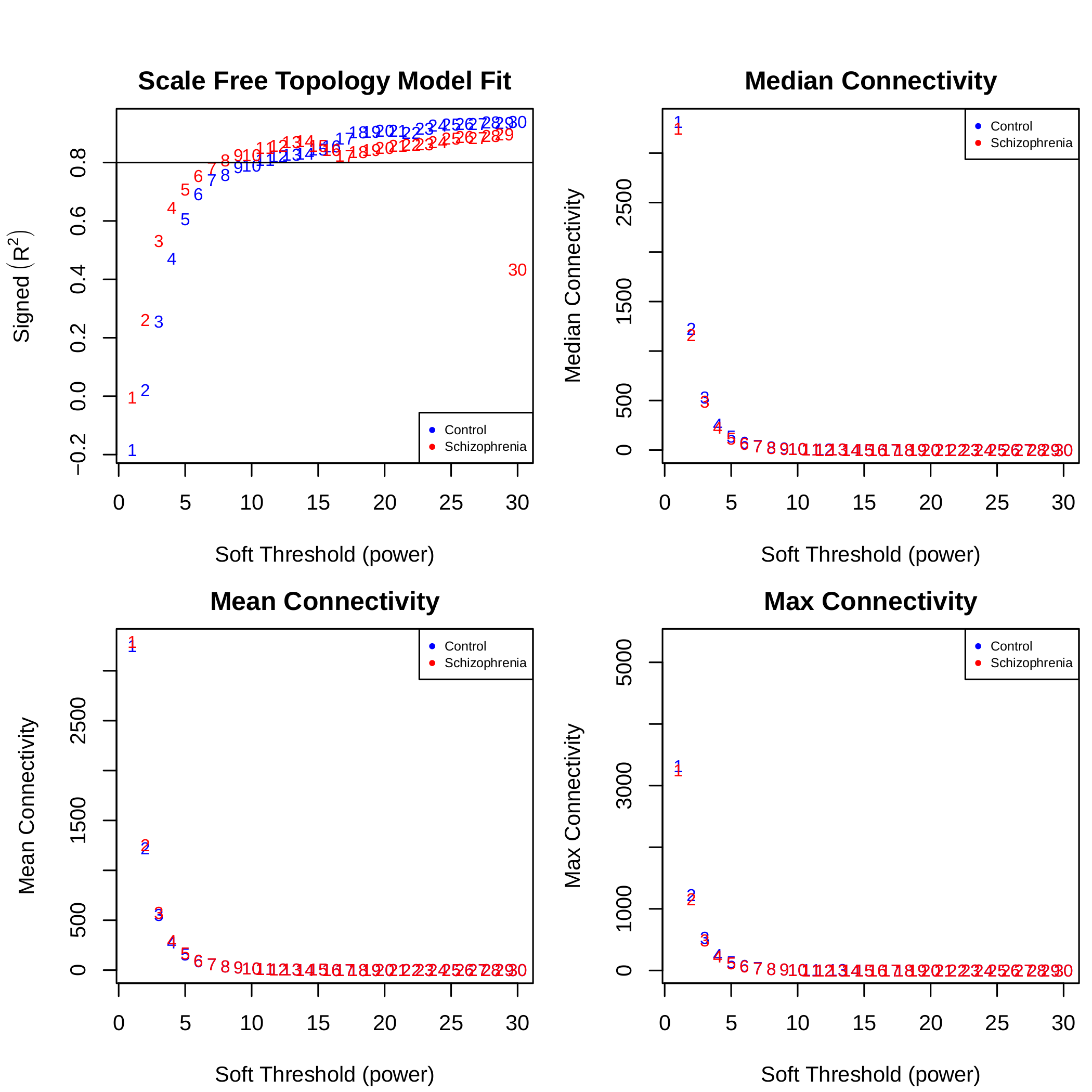

Supplement: S3 Fig — In all subfigures, each number represents a different power (or β value) for both control (blue color) and schizophrenia (red color) networks. For both networks, the β value chosen was 14. (TIFF) [file pone.0210431.s003.tiff]

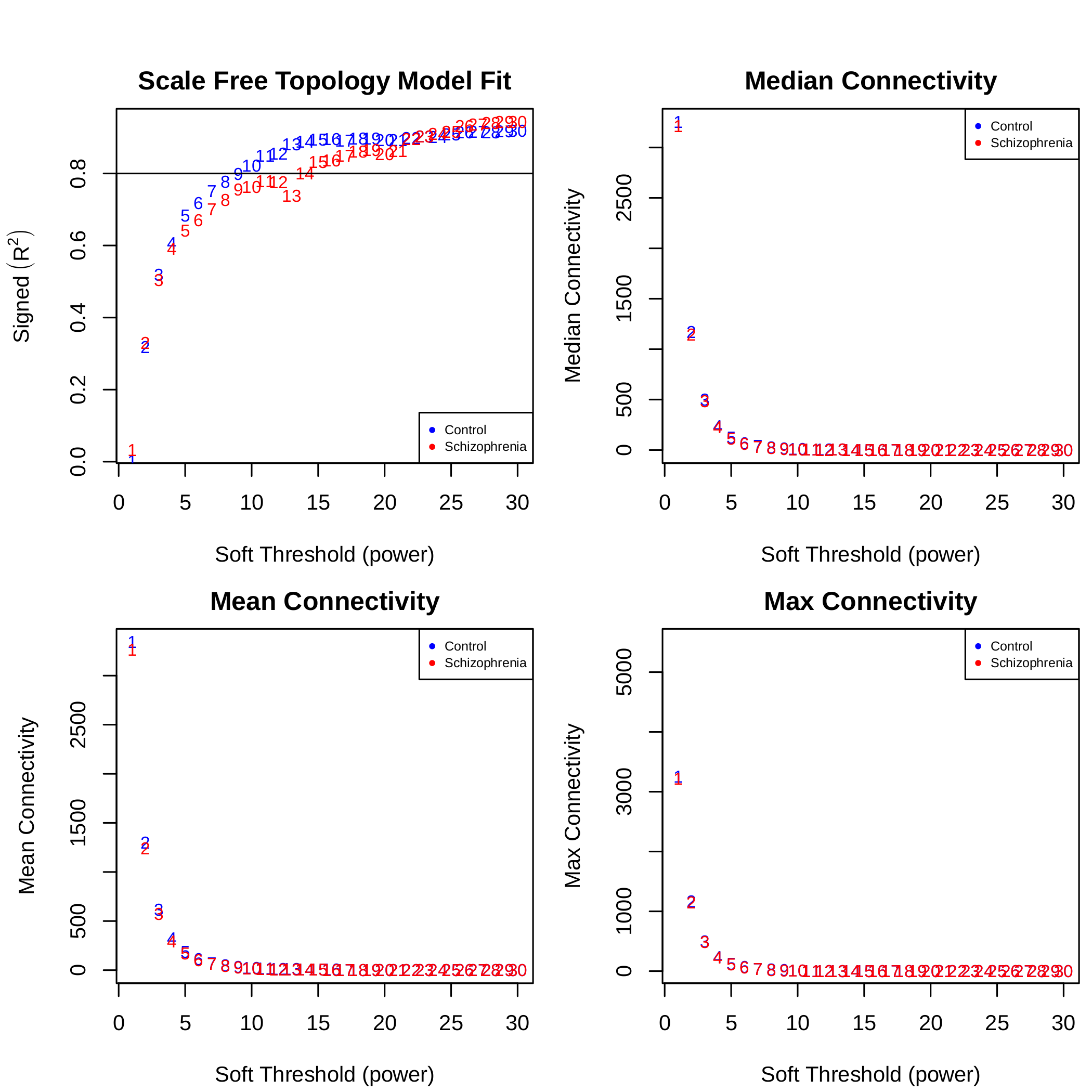

Supplement: S4 Fig — In all subfigures, each number represents a different power (or β value) for both control (blue color) and schizophrenia (red color) networks. For both networks, the β value chosen was 14. (TIFF) [file pone.0210431.s004.tiff]

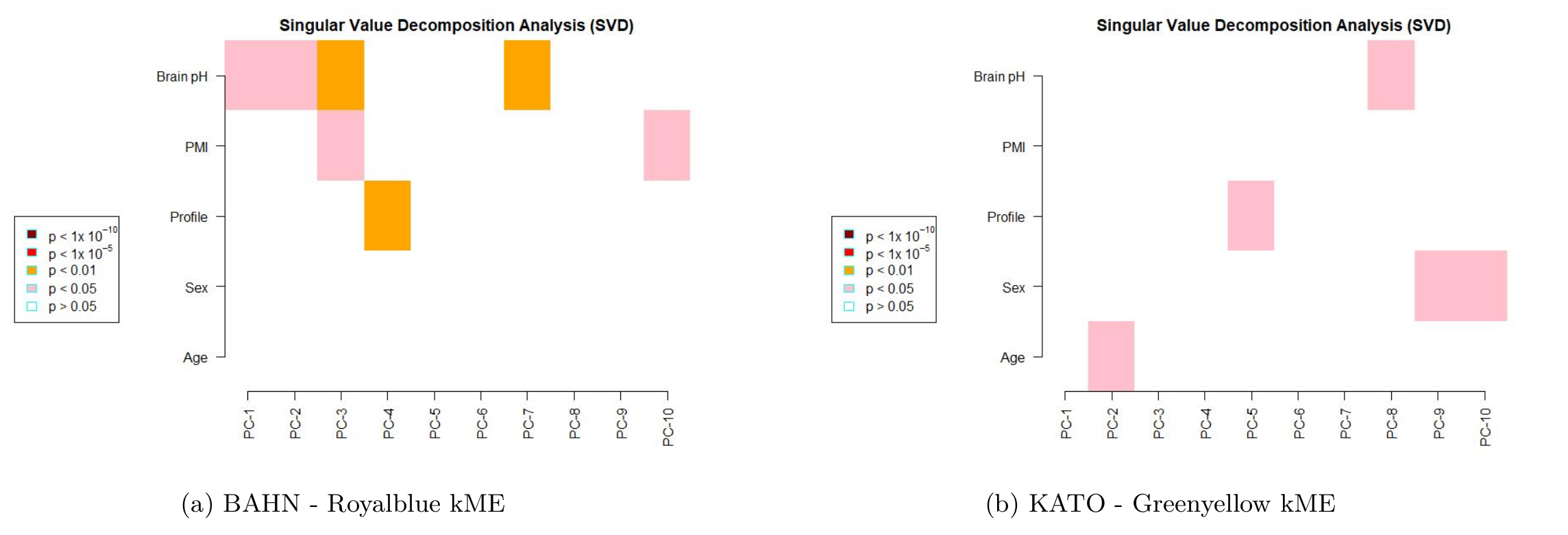

Supplement: S5 Fig — On (a): clustering dendogram based on euclidian distance (y axis) of the control and schizophrenia samples; (b): Greenyellow kME pearson correlation with the expression values of all 365 genes contained on Royalblue Module control group (x axis) and schizophrenia group (y axis) with corp = 0.5. (TIFF) [file pone.0210431.s005.tiff]

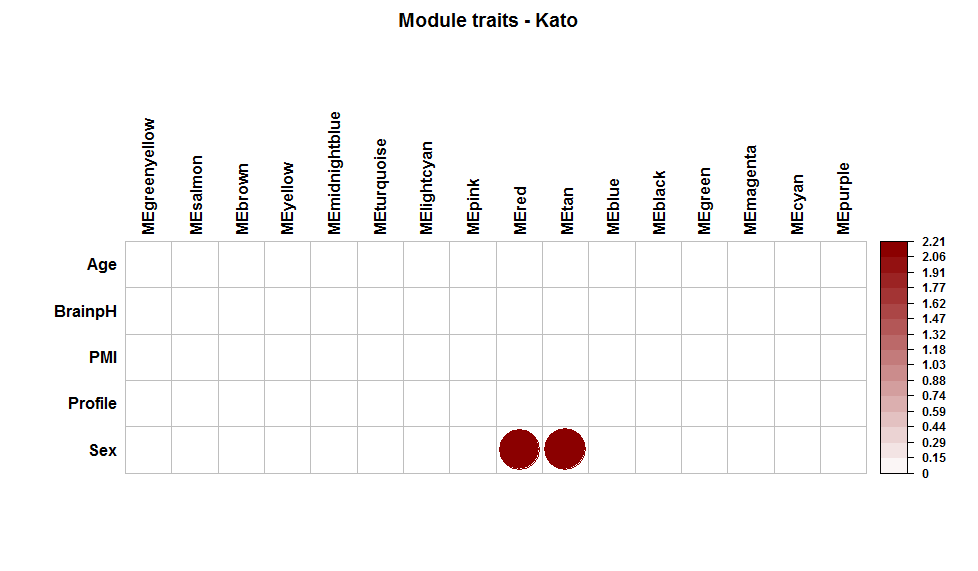

Supplement: S6 Fig — Age, Brain pH, PMI, Disease status (Profile) and Gender (sex) are represented on the y-axis. The p-values are represented on a -log10 scale. (TIFF) [file pone.0210431.s006.tiff]

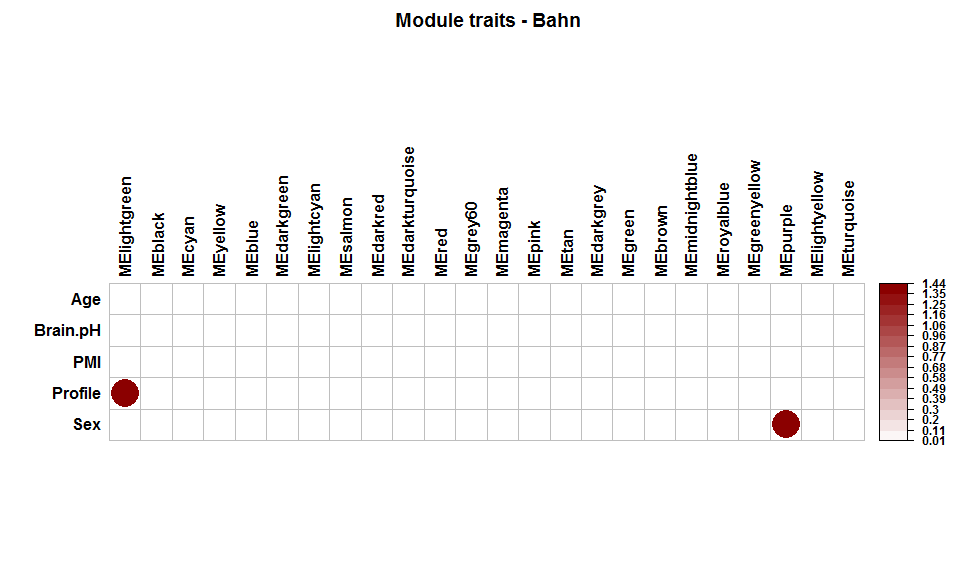

Supplement: S7 Fig — Age, Brain pH, PMI, Disease status (Profile) and Gender (sex) are represented on the y-axis. The p-values are represented on a -log10 scale. (TIFF) [file pone.0210431.s007.tiff]
